# Noninvasive targeting delivery and in vivo magnetic resonance tracking method for live apoptotic cells in cerebral ischemia with functional Fe_2_O_3_ magnetic nanoparticles

**DOI:** 10.1186/s12951-016-0173-1

**Published:** 2016-03-11

**Authors:** Atsushi Saito, Moataz M. Mekawy, Akira Sumiyoshi, Jorge J. Riera, Hiroaki Shimizu, Ryuta Kawashima, Teiji Tominaga

**Affiliations:** Department of Neurosurgery, Aomori Prefectural Central Hospital, 2-1-1 Higashitsukurimichi, Aomori, 030-8553 Japan; Department of Neurosurgery, Graduate School of Medicine, Tohoku University, 2-1 Seiryo-machi, Aoba-ku, Sendai, Miyagi 980-8575 Japan; Department of Functional Brain Imaging, Institute of Development, Aging and Cancer, Tohoku University, 2-1 Seiryo-machi, Aoba-ku, Sendai, Miyagi 980-8575 Japan; Department of Neurosurgery, Graduate School of Medicine, Akita University, 1-1-1 Hondo, Akita, 010-8543 Japan; National Institute for Materials Science, 1-Chome-2-1 Sengen, Tsukuba, Ibaraki Prefecture 305-0047 Japan

**Keywords:** Apoptosis, Functionalized magnetic nanoparticles (FMNPs), Magnetic resonance imaging (MRI), Fluorescently-labeled poly-caspase inhibitor (SR-FLIVO)

## Abstract

**Background:**

Apoptotic neuronal death is known as programmed cell death. Inhibition of this progression might contribute to a new treatment strategy. However, methods for in vivo detection of live apoptotic cells are in need to be developed and established.

**Context and purpose:**

The purpose of this study is to develop a new method for in vivo brain imaging for live apoptotic lesions using magnetic resonance imaging (MRI). We focused on the specific accumulation of our recently developed functional magnetic nanoparticles (FMNPs) into apoptotic cells using a rat cerebral ischemia model. Sulphorhodamine B, fluorescent dye was linked to valylalanylaspartic acid fluoromethyl ketone as a pan-caspase inhibitor to form SR-FLIVO. SR-FLIVO was bound with FMNPs to develop SR-FLIVO-FMNP probe. Ischemic rat brains were scanned by 7T MRI before and after intravenous injection of SR-FLIVO-FMNP and the distribution was evaluated by subtraction images of T2* colored mapping. SR-FLIVO, intracellular FMNPs, and T2* reduction area were histologically analyzed. The distribution of SR-FLIVO-FMNP was evaluated by subtracting the T2* signal images and was significantly correlated with the histological findings by TUNEL staining.

**Results:**

Our experimental results revealed several findings where our newly developed probe SR-FLIVO-FMNP was intravenously administered into ischemic rats and FLIVO expression was tracked and found in apoptotic cells in rat brains after cerebral ischemia. A remarkable T2* reduction within the ischemic lesion was recorded using MRI based SR-FLIVO-FMNP probe as a contrasting agent due to the specific probe accumulation in apoptotic cells whereas, no observation of T2* reduction within the non-ischemic lesion due to no probe accumulation in non-apoptotic cells. Histological analysis based on the correlation between FLIVO and TUNEL staining showed that almost all FLIVO-positive cells were positive for TUNEL staining. These findings suggest the possibility for establishment of in vivo targeting delivery methods to live apoptotic cells based on conjugation of magnetic and fluorescent dual functional probes.

**Conclusion:**

A newly developed probe SR-FLIVO-FMNP might be considered as a useful probe for in vivo apoptotic detection, and FMNPs might be a strong platform for noninvasive imaging and targeting delivery.

**Electronic supplementary material:**

The online version of this article (doi:10.1186/s12951-016-0173-1) contains supplementary material, which is available to authorized users.

## Background

Apoptosis plays a key role in the pathogenesis of a variety of disorders including cerebral and myocardial ischemia, autoimmune and neurodegenerative diseases, infections, organ and bone marrow transplant rejection, and tumor response to chemotherapy and/or radiotherapy [1, 2]. At present, non-invasive techniques for direct in vivo detection of apoptotic cells are quite rare and urgently need improvement. Early in vivo detection of apoptotic cells can provide the physician with important information to develop further therapeutic strategies in chemotherapy or radiotherapy of tumors, in transplantation of organs, or in rescue of ischemic areas [2]. However, there are few, if any, reagents are currently available to assess the occurrence of apoptosis in living tissue.

Previously, several attempts had been carried out to detect apoptosis using different imaging techniques. Via microscopy, Zhang et al. [3] reported the conjugation of Annexin V with polyethylene glycol-coated core cross linked polymeric micelles (CCPMs) labeled with (^111^In) to form A5-CCPM for dual SPECT and optical microscopic detections. However, short blood life time of Annexin A5 makes the time point of assessment very critical to be monitored. Meanwhile, the inversion of phosphatidyl-serine may not be exclusively related to apoptosis and this adds to the background issues [4–7]. Yivgi-Ohana et al. [8] used fluorescence microscopy to examine the in vivo apoptosis expression of split yellow fluorescence protein (YFP) fragments in liver hepatocytes.

In clinical diagnostics, MRI is considered as the most powerful non-invasive imaging tool. Since MRI technique has a relatively higher spatial resolution than previously reported techniques such as magnetic resonance spectroscopy (MRS) and radionuclide techniques, thus; it could show better detection for apoptotic lesions aided with suitable contrasting agent [9, 10]. The key features for apoptosis detection mainly depend on the detection of phosphatidylserine derivatives or activated caspases. Several studies reported the usage of different probes as MRI contrasting agents to detect apoptosis based on the conjugation of these developed probes with phosphatidylserine derivatives located on the surface of the cell membrane [11–13].

We focused on a new fluorescent dye-conjugated apoptotic detector, FLIVO (fluorescence in vivo). This effective moiety is composed of a polycaspase inhibitor, SR-Val-Ala-Asp-fluoromethyl ketone (SR-VAD-FMK), and sulphorhodamine B. SR-VAD-FMK is a well-known inhibitor that irreversibly binds to the catalytic site of caspase proteases and can be delivered specifically into apoptotic neuronal cells intravenously after stroke [14–16]. FLIVO has been shown to have characteristics that are useful for an in vivo apoptotic marker, such as general solubility, cell permeability into the blood–brain barrier (BBB), long plasma circulation (30–60 min) and the ability to form covalent bonds with the active site of the cysteine-dependant aspartate-specific protease (caspases) enzyme. In addition, the free or unbound probe is readily removed from cells [14–17].

Nanotechnology, as it relates to biomedicine, can be broadly defined as nano-sized structures that have at least one dimension between 1 and 100 nm and exhibit new or enhanced properties that are unattainable at both smaller and larger levels [18]. The therapy and diagnosis (theranostics) of pathologies that affect the central nervous system are currently undergoing a renaissance because of the marked proliferation of nanoscale technologies [19]. Several nanomaterials have been used as platforms for the theranostics of stroke showing promising research results that could be applicable in future clinical applications once completed and approved [20, 21]. USPIO are based on magnetite, which has received much attention for biomedical applications, or maghemite molecules encased in polysaccharide, synthetic polymers, or monomer coatings [20–22].

The utility of magnetic iron oxide nanoparticles (MNPs) as a magnetic resonance imaging (MRI) contrast agent has been studied for more than two decades [18, 19]. Iron oxide MNPs that possess a diameter between 10 and 50 nm are classified as ultra-small superparamagnetic particles of iron oxide (USPIO). USPIO is known to be permeable to the BBB and are taken up by monocytes and macrophages after intravenous administration [19–22]. USPIO tend to be applied more in magnetic susceptibility-based acquisitions in T2- or T2*-weighted MRI, in which they produce a hypointense signal [19].

There is a great deal of interest in developing multimodality probes for molecular imaging [22–24]. Magnetic iron oxide nanoparticles have been widely studied for biological and biomedical applications owing to their useful magnetic properties [25, 26]. MNPs effectively reduce the proton relaxation time in MRI and hence, lead to decreases in T1, T2, and T2* quantities [22]. It should be noted that the transverse relaxivity (T2 and T2*) of MNP contrast agents is far greater than their longitudinal relaxivity (T1), which explains why MNPs are used mainly as negative agents for T2/T2*-weighted imaging [27, 28].

In this study, we aimed to develop a new method for in vivo targeting delivery for live apoptotic lesions in the ischemic brain and for image tracking by MRI. For the first time, a multifunctional apoptotic detecting probe, SR-FLIVO-FMNP, was fabricated by binding sulphforhodamine B, fluorescent dye-conjugated FLIVO, with functionalized Fe_2_O_3_ MNPs. In vivo apoptotic neuronal cell death in ischemic rat brains was induced by an established transient focal cerebral ischemia (tFCI) model [29, 30]. We report the effectiveness and promise of this new probe of live neuronal apoptotic cells in rat brains after cerebral ischemia using MRI.

## Methods

### Fabrication of SR-FLIVO-FMNP

Synthesis of Fe_2_O_3_ MNPs, surface fictionalization and conjugation to SR-FLIVO was carried out as previously described [31]. Breifly, 5.0 g of 0.15 mM FeCl_2_·4H_2_O was mixed with 0.5 mL of 1.0 M HCl at room temperature for 5 min. to form solution A. 1.2 g CTAB was dissolved with 10 mL *n*-Octane followed by addition of 10 mL 1-butanol and stirring at 40 °C for 20 min to form solution B. Solutions A and B were mixed together under vigorous stirring for 30 min at 40 °C followed by addition of 6.0 mL of 0.25 M NaOH and kept under stirring for another 20 min to produce microemulsion of Fe_2_O_3_ nanoparticles. Decantation and then centrifugation carried out and finally the NP supernatant was washed thoroughly with water and acetone and kept for drying.

For silica-shell coating iron oxide core, a starting sol contains an equi-molar of Na_2_SiO_3_·9H_2_O:FeCl_2_·4H_2_O as silica and iron sources, respectively was used under the same mentioned conditions. Removal of the hexadecyltrimethylammonium bromide (CTAB) surfactant was carried out using solvent extraction method where NPs (Fe_2_O_3_ and Fe_2_O_3_–SiO_2_) were immersed in hot ethanol at 50 °C for 20 h. Finally the surface grafting with -NH_2_ group for Fe_2_O_3_–SiO_2_ NPs was carried out using 3-Aminopropyltriethoxysilane (APTES) by immersing 1.0 g of the NPs in 20 % APTES/Toluene mixture and allowed to interact for 12 h at 50 °C followed by washing thoroughly with ethanol and hexane. Formation of SR-FLIVO-FMNP was carried out using 1.0 mg of FNPs/1.0 mL of SR-FLIVO.

### Rat cerebral ischemia model

Animal experimental procedures were conducted according to the guidelines established and approved by Animal Care Committee at Tohoku University. Animal arrive guidelines were followed in the preparation of the manuscript. Adult male rats (3 months old, 260–290 g) were subjected to tFCI by intraluminal MCA blockade with a nylon suture as described previously [30]. The rats were anesthetized with 1.5 % isoflurane in 30 % oxygen and 70 % nitrous oxide using a face mask. The rectal temperature was controlled at 37 °C. Blood gas was analyzed with a pH/blood gas analyzer (Chiron Diagnostics Ltd). After a midline skin incision, the left external carotid artery was exposed and its branches were electrocoagulated. A 22.0 mm 3–0 surgical monofilament nylon suture, blunted at the end, was introduced into the left internal carotid artery through the external carotid artery stump. After 90 min of middle cerebral artery occlusion, blood flow was restored by the withdrawal of the nylon suture. For probe (bare FMNP or SR-FLIVO-FMNP, *n* = 4 each) administration, the left jugular vein was exposed between the sternocleidomastoid muscle and anterior perusal muscle. 100 μg of SR-FLIVO were diluted in 50 μl of DMSO followed by addition of 200 μl of PBS buffer. The volume ratio of DMSO/PBS buffer was kept 1:5. The left jugular vein was cannulated with a 23–gauge needle and 600 μl of SR-FLIVO-FMNP or bare FMNP/PBS (1.0 mg/ml) solution was injected and kept in blood circulation for 60 min before MRI signal recording.

### Histochemical analyses

Methods of staining and immunohistochemical assessment were according to the previously described in Saito et al. [1] Anesthetized animals, as well as normal controls (*n* = 4 each), were perfused with 10 U/ml heparin and subsequently with 4 % paraformaldehyde in 0.1 M PBS (pH 7.4) at 8, 12 and 24 h of reperfusion. The brains were removed, post-fixed for 12 h and sectioned to 50 μm using vibratome (Leica VT1000S). Immunohistochemistry was performed using the avidin–biotin technique and then the nuclei were counterstained with methyl green solution for 2 min, or fluorescein isothiocyanate or Texas Red-conjugated secondary antibody. For histological assessment, alternate slices from each brain section were stained with hematoxylin and eosin or cresyl violet.

### Histological detection of apoptosis

To clarify the spatial distribution of apoptotic cells after cerebral ischemia, we performed terminal deoxynucleotidyl transferase-mediated uridine 5′-triphosphate-biotin nick end labeling (TUNEL) staining as previously described (*n* = 8 each) [1]. Fixed sections were incubated with NeuroPore (Trevigen) for 30 min. They were placed in 1 × terminal deoxynucleotidyl transferase (TdT) buffer (Invitrogen) with a TdT enzyme (Invitrogen) and biotinylated 16-dUTP (Roche Diagnostics) at 37 °C for 90 min. The avidin–biotin technique was applied and then the nuclei were counterstained with methyl green solution for 2 min. For more precise confirmation about SR-FLIVO-FMNP probe tracking within the apoptotic cells, high resolution transmission electron microscopy (HRTEM) was recorded for the cells located at ischemic and non-ischemic lesions.

### MRI measurements

Each rat was initially anesthetized with 5 % isoflurane. All MRI data were acquired using a 7T Bruker PharmaScan system (Bruker Biospin, Ettlingen, Germany) with a 38-mm-diameter birdcage coil. T2* mappings were obtained using a respiratory-gated multiple gradient echo (MGE) imaging sequence with the following parameters: TR = 1500 ms, TE = 4, 11, 18, 25, 32, 39, 46, 53, 60, 67, 74, and 81 ms, flip angle = 20°, field-of-view = 32 × 32 mm^2^, matrix size = 200 × 200, in-plane resolution = 160 × 160 um^2^, slice thickness = 1 mm, slice gap = 0 mm, number of slices = 16, and number of averages = 4. T2-weighted anatomical images were obtained using a respiratory-gated 2D TurboRARE sequence with fat suppression under the following parameters: TR = 4000 ms, TEeff = 36 ms, RARE factor = 8, flip angle = 90°, field-of-view = 32 × 32 mm^2^, matrix size = 200 × 200, in-plane resolution = 160 × 160 um^2^, slice thickness = 1 mm, slice gap = 0 mm, number of slices = 16, and number of averages = 10. MGE images were analyzed using custom-written software, MATLAB (R2009b; MathWorks). The analytical procedures have been previously described [32]. T2* reduction maps were derived from the simple T2* value subtraction in a voxel-by-voxel manner (i.e., T2*_post inj._–T2*_pre inj._). The MRI phantom was constructed from 1 mL plastic syringes that contained different concentrations of the SR-FLIVO-FMNP sample (0.25–2 mg/mL in PBS) and 1 % gadoterate meglumine (Magnescope, Terumo, Tokyo, Japan) as a standard of comparison. The MRI parameters of phantom scanning were exactly the same as above.

### Statistical analysis for cell counting and T2* signal intensity

ROIs in an area of 5 × 5 mm^2^ were determined on histological sections collected from the three studied groups (control, FMNP and SR-FLIVO-FMNP injected rats). SR-FLIVO-positive cells and TUNEL-positive cells were counted and the immunopositive ratios were calculated. The anatomically appropriate slice in scanned MRIs was selected and the voxels were determined according to the ROIs on the histological sections. Averaged reduction values in T2* signal intensity in each voxel were calculated and compared with the immunopositive ratios. Correlations of these values were analyzed with Pearson correlative analysis and *P* values were calculated.

## Results and discussion

### Formation of SR-FLIVO-FMNP

**Fig. 1:**
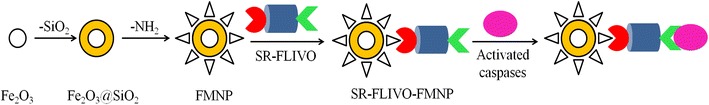
A schematic illustration of the proposed formation mechanism of the core Fe_2_O_3_ MNP at SiO_2_ shell with −NH_2_ group-fictionalization to form FMNP and conjugation to SR-FLIVO to form SR-FLIVO-FMNP probe that is selectively interacting with activated caspases

A representative scheme (Fig. 1) is elucidated for the formation mechanism of FMNP and its conjugation to SR-FLIVO to form SR-FLIVO-FMNP probe. Characterization using TEM, Fluorescence spectrophotometer and FTIR showed that; the average size of FMNP was determined to be 11.6 ± 0.6 nm and its surface could be successfully conjugated to SR-FLIVO with 80 % binding efficiency [31].

### MRI signal intensity of SR-FLIVO-FMNP

**Fig. 2:**
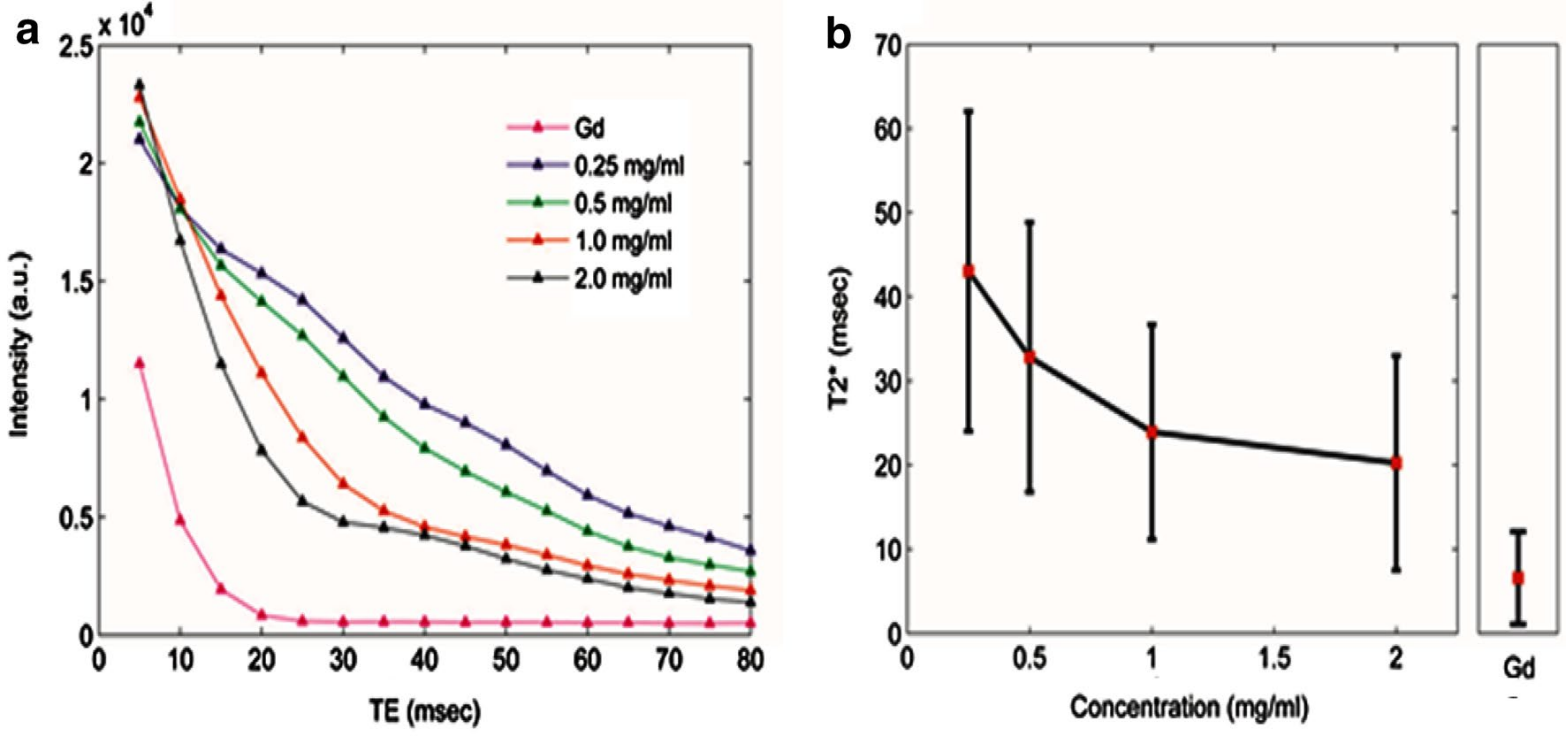
The concentration dependent results of the phantom MRI measurements (**a**). The averaged T2* quantities of SR-FLIVO-FMNP with standard deviation. *Gd* 1 % gadoterate meglumine (**b**)

To evaluate the magnetic property of the conjugated probe, we performed a phantom MRI experiment with SR-FLIVO-FMNP and 1 % gadoterate meglumine (Fig. 2). As expected, the higher concentration of SR-FLIVO-FMNP showed relatively faster decay of MR signal intensity, whilst the lower concentration showed a slower decay of MR signal intensity. This result verified that the newly synthesized SR-FLIVO-FMNP probe possesses the magnetic contrast property in a concentration-dependent manner. It should be noted that the influence of SR-FLIVO-FMNP on T2* reduction was relatively modest compared with that of a standard or commercially available contrast agent based on Fe_3_O_4_ MNPs (Fig. 2). Since Fe_2_O_3_ is the most stable compound among the iron oxides and all other iron oxide forms get converted to Fe_2_O_3_ easily. Thus, it is expected to have a phase change due to the presence of reactive oxygen species accompanying apoptosis. However, the reported r2 of bare core Fe_3_O_4_ MNPs with comparable size is 198 mM^−1^ S^−1^ whereas the estimated r2 for our bare core Fe_2_O_3_ MNPs is 42.8 mM^−1^ S^−1^ (Additional file 1). A higher effect of core Fe_3_O_4_ on T2 and T2* reduction could be expected and ascribed due to its higher magnetization radius than Fe_2_O_3_ [33, 34].

### Histological detection of apoptotic cells in rat brains after cerebral ischemia

**Fig. 3:**
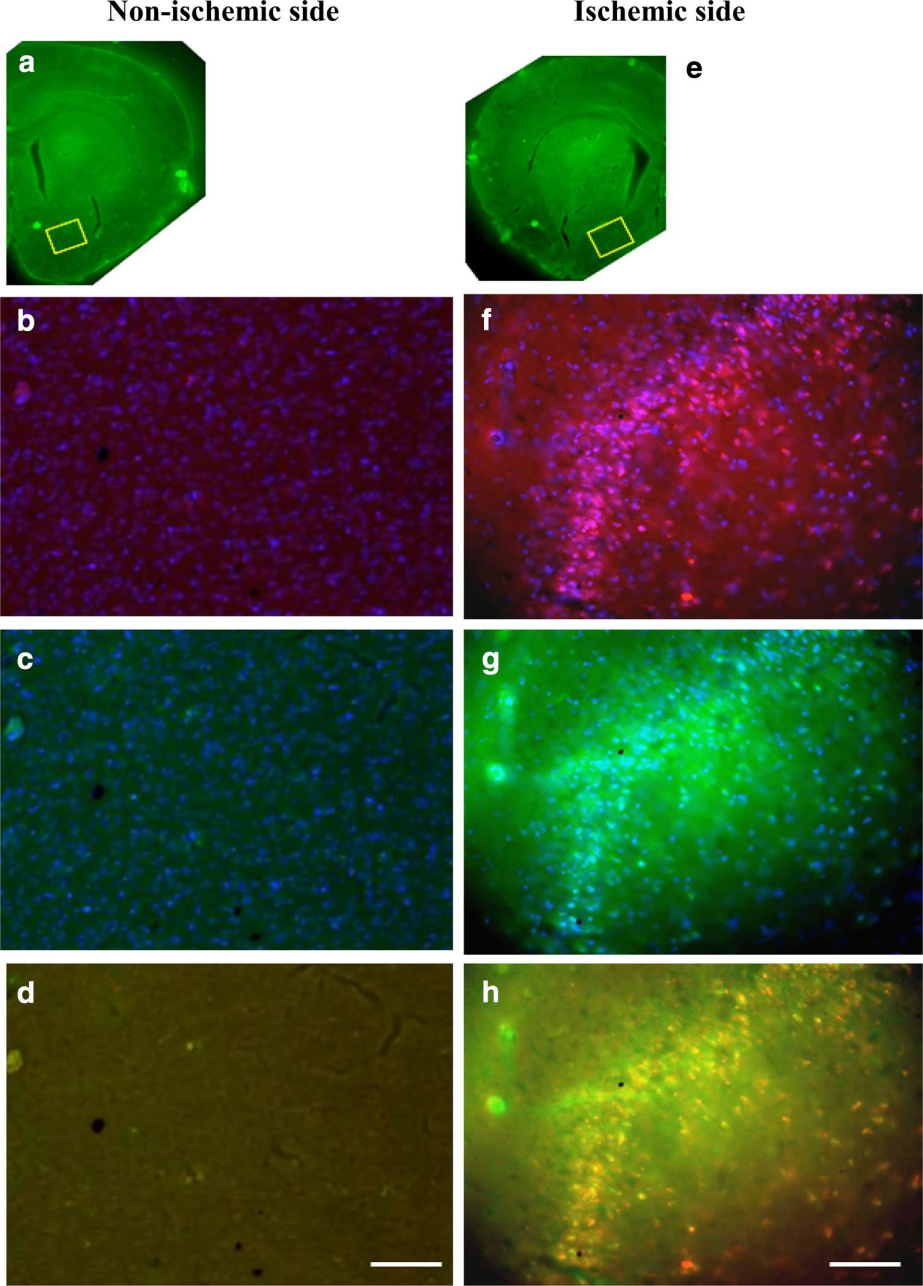
Representative photographs of fluorescent images of SR-FLIVO (*red*, **b**, **f**) and TUNEL staining (*green*, **c**, **g**) on the non-ischemic side (**a**–**d**) and the ischemic side (**e**–**h**). The ROI is set in *yellow squares* in the non-ischemic area (**a**) and the ischemic area (**e**). *Merged* images of SR-FLIVO and TUNEL staining are shown in **d** and **h**. Almost no positive fluorescent expression was observed on the non-ischemic side (**a**–**d**). SR-FLIVO expression (**f**) and TUNEL immunopositivity (**g**) were observed in the region around the ischemic penumbra. Intracellular co-localization of these two immunopositivities can be detected in the merged image (*orange*, **h**). *Bars* 100 μm

Our study group has reported that TUNEL staining showed immunopositive cells in the penumbra region of ischemic rat brains within 24 h after reperfusion injury [30]. Intravenous administration of SR-FLIVO-FMNP induced intracellular accumulation of SR-FLIVO in apoptotic TUNEL-positive cells in the ischemic penumbra (Fig. 3).

TUNEL has arguably been the most common labeling method used for the identification of apoptotic cells. However, TUNEL cannot be used for living cells or animals since it requires permeabilization of the cell membrane [14, 35, 36]. What has been attempted for imaging of apoptotic activity in patients or animals has typically involved radioactive labeling with annexin V and autoradiography or other complex imaging strategies using MRI [37, 38]. Annexin V has a benefit as an early marker of programmed cell death however, due to its very short plasma half life time (<5 min.), it is difficult to reliably detect its interaction since the time point of assessment can be critical. In addition, annexin V does not bind to all apoptotic tumor cells, and it also binds positively to normal and healthy bone marrow-derived cells [39– 41]. Furthermore, the inversion of phosphatidyl-serine may not be exclusively related to apoptosis [6, 7]. On the other hand, our developed probe has been allowed to be under blood circulation for 60 min. before MRI recording and our results confirmed existence of SR-FLIVO positivity within the ischemic lesion.

The proposed mechanism of the process could be explained based on the activation of caspases apoptosis, led to having a cysteine residue of these proteases as their active centers. SR-FLIVO as a main component in our probe has three segments; an inhibitor, which is selectively binding the active center of the caspase; a reactive fluoromethylketone (FMK) segment which links irreversibly the thio-methyl ketone with the cysteine at the active center of caspase; and red sulforhodamine B segment as a fluorophore which helps in fluorescence detection of the cells that bound to the porbe. Selective binding could occur with the active rather than inactive caspases taking into account that they are labeling apoptotic cells. FLIVO includes the peptide-based, poly caspase-binding– inhibitor probe [Val-Ala-Asp(OMe)], which contains an O-methylation moiety in the Aspartic residue that provides enhanced stability and cell permeability to the inhibitor. Since SR-FLIVO is cell permeant, thus; they can be i.v. injected into living rats, in which they can selectively bind to cells displaying active caspases, resulting in the trapping of the red SR-FLIVO fluorescence signal within these cells. FLIVO can’t give positive expression with non-apoptotic cells due to its leakage outside the cells that lack caspase activity [42].

### MRI of in vivo apoptosis in ischemic rat brains

**Fig. 4:**
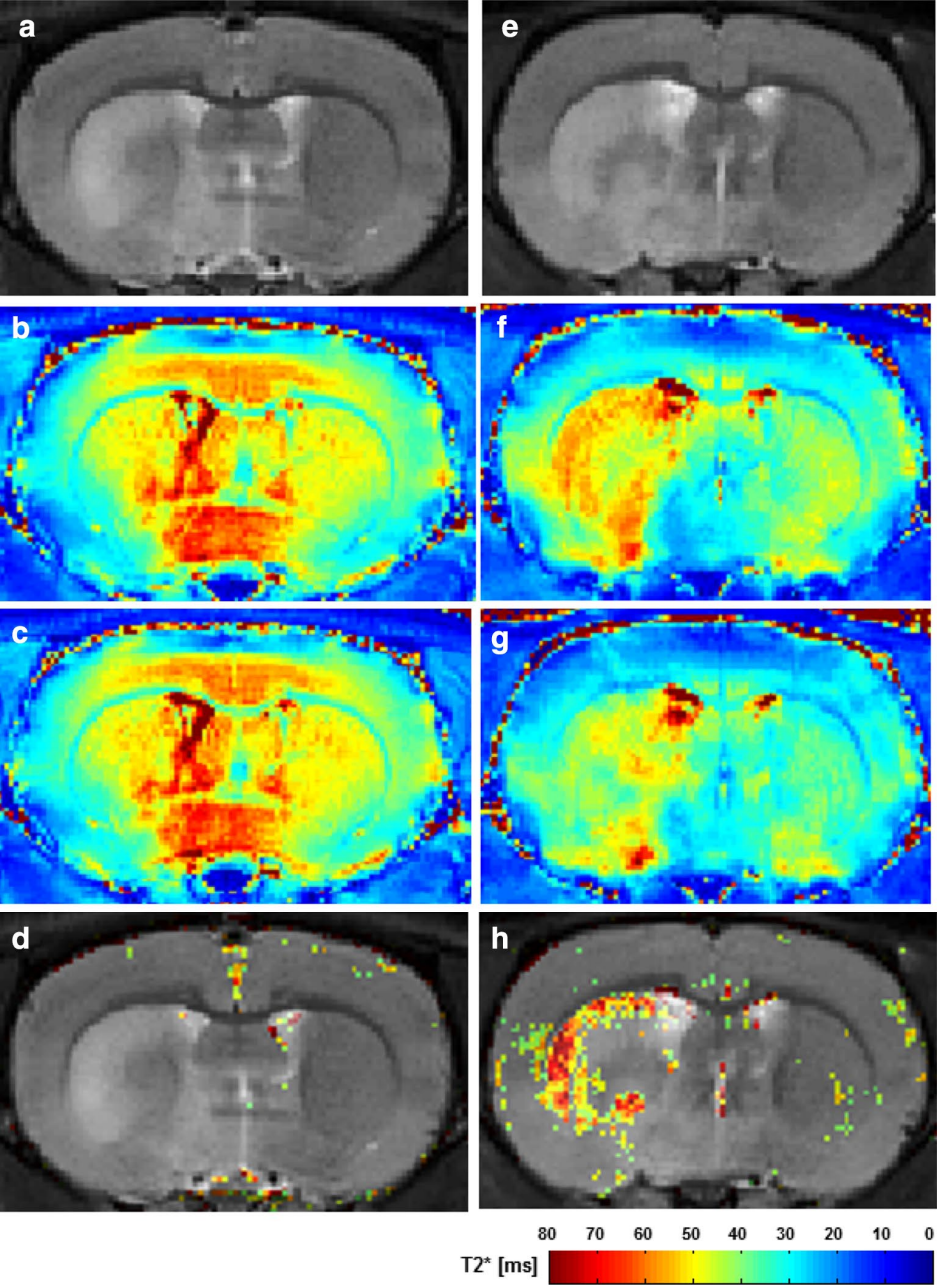
Representative magnetic resonance images of ischemic rat brains administered with bare FMNPs (**a**–**d**) and with SR-FLIVO-FMNPs (**e**–**h**). T2-weighted images (**a**, **e**), T2*-mappings of pre-injection (**b**, **f**), T2*-mappings of post-injection (**c**, **g**), and overlaid images of subtraction between before and after injection on T2-weighted images (**d**, **h**). There was no signal change between pre- and post-injection of bare MNPs (**b**, **c**). T2* signal reduction was prominently observed after injection of SR-FLIVO-FMNPs (**f**, **g**)

A T2-weighted image revealed high intensity in the caudate putamen area supplied by the left MCA 24 h after ischemic reperfusion injury. SR-FLIVO-FMNP was intravenously injected and T2* mapping was scanned before and after the injection. Superparamagnetic iron oxide nanoparticles (SPION) was reported to show hypointensity in T2* mapping in rodent brains in vivo [20, 21, 23]. Vascular-rich tissue such as choroid plexus showed T2* reduction induced by a slight non-specific uptake of iron nanoparticles as previously reported [43]. By subtracting images between pre- and post-injection, we calculated a T2* reduction area in the ischemic lesion without a non-specific uptake of iron particles in the background, as previously reported [44]. A T2* reduction region was observed around the ischemic core adjacent to the cortical area in ischemic rat brains after SR-FLIVO-MNP injection. No recorded T2* signal reduction was shown in bare FMNP-injection without conjugation of SR-FLIVO (Fig. 4).

MRI monitoring of molecular events in live cells in rodent brains has been previously reported. Varallyay et al. [45] reported many promising results from the application of MNPs for MRI monitoring of a rat brain tumor model. Their group intravenously injected USPIO as passive targeting of a lesion that destroyed the BBB, as induced by a brain tumor, and detected iron oxide accumulation by 12T dynamic contrast-enhanced MRI fabricated superparamagnetic iron oxide nanoparticles (SPION)-glial fibrillary acidic protein (SPION**-**GFAP) probe for gene targeting in live brains. They administered SPION-GFAP via the conjunctiva sac in rat cortical spreading depression models. SPION-GFAP was successfully accumulated in the β-actin-expressing region after permeation into the BBB and was detected by MRI as a T2* signal reduction region. These reports suggest the usefulness of MNPs for molecular MRI monitoring, and the future development of active targeting technology is strongly anticipated.

Surface-modified iron oxide MNPs could behave as contrast agents for facile targeting in MRI [46]. The biocompatibility of MNPs could be enhanced after careful study of the surface structures. This could allow facile shielding of the magnetic core with bio-polymers, which may lead to some extent to aggregation of the NPs, giving final products of larger sizes, or an inorganic silica shell that allows facile grafting to the surfaces and gives more stability to the MNP core against biodegradation when using various solvent types and/or under physiological conditions [47]. Our new molecular design has been fabricated on the basis of the conjugation between the Fe_2_O_3_ MNP core grafted with thin silica shell functionalized with an amine group-NH_2_ from one side and SR-FLIVO as a caspase inhibitor from the other side. To our knowledge, this is the first study to address such a molecular design for the biomedical application for diagnosis of brain apoptotic lesions arising from brain transient cerebral ischemia. Bare Fe_2_O_3_ MNPs can be expected to have higher nano-toxicity however; surface coating of magnetic core using ad-layer synthesis led to having a shield composed of hybrid inorganic–organic moieties (SiO_2_, −NH_2_ and SR-VAD-FMK) that are enhancing the stability and biocompatibility of the designed SR-FLIVO-FMNP probe as a whole which possess slightly negative charge. (Additional file 1: Figure S2 represents the surface zeta potential of SR-FLIVO-FMNP probe which shows a sightly negative surface charge). Several studies demonstrated that; NPs with mixed chemical structures have shown high compatibility including low hemolytic rates less than the cut-off limit (<5 %) increasing the opportunity of their application for in vivo cellular tracking and imaging [48]. Nevertheless, Mocan et al. [49] reported that; apoptotic cells are recognized by macrophages equipped with receptors specific for phosphatidylserine such as Annexin V. The macrophages swoop and degrade phosphatidylserine-exposing cells. Therefore, apoptosis allows the elimination of those cells without releasing of their intracellular proteins which could lead to inflammation. Contrary, erythrocytes lack nuclei and mitochondria which are the main in the apoptosis mechanism. Thus; the death of erythrocytes were considered to be eliminated by other mechanism nominated as eryptosis. Symptoms of both mechanisms could be quite similar however, apoptosis in erythrocytes is generally regarded as a mechanism that inhibits hemolysis and leads to a temporary stabilization of cells.

### Histological evaluation of the T2* reduction region on MRI

**Fig. 5:**
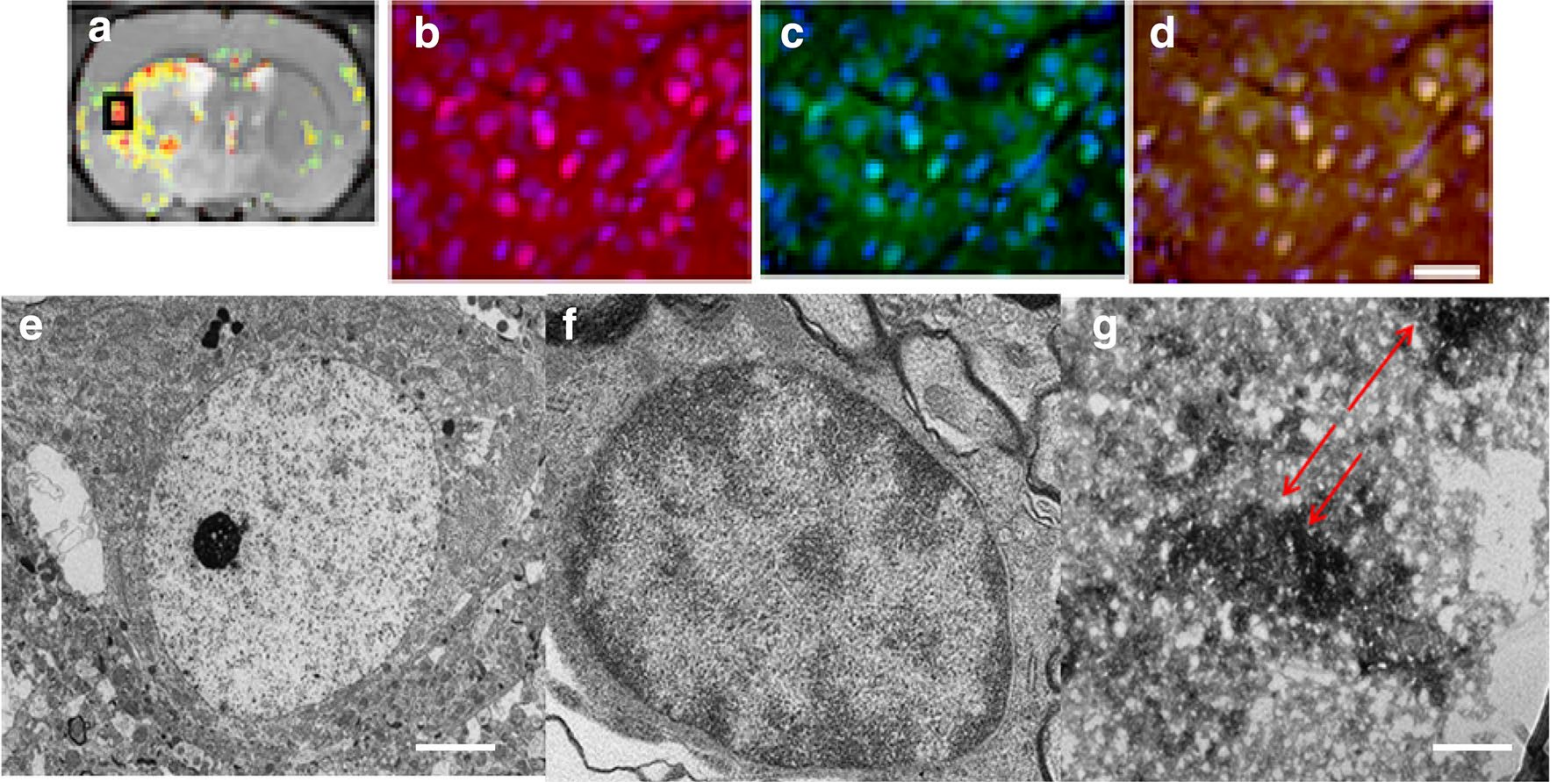
The ROI is set in a *black square* in the T2* signal reduction region (**a**). Fluorescent images of the ischemic brain section in the targeting area (**b**–**d**). SR-FLIVO expression (**b**) and TUNEL immunopositivity (**c**) were co-localized as shown in the *merged image* (**d**). *Bar* 20 μm. **e**–**g** Electron micrographs demonstrated no localization of SR-FLIVO-FMNP probe inside control cell in non-ischemic core, whereas, it was localized within apoptotic cell in the ischemic core. *Arrows point* to the FMNP conjugated SR-FLIVO (**g**). *Bar* 500 nm for **e** and **f** and 100 nm for **g**

**Fig. 6:**
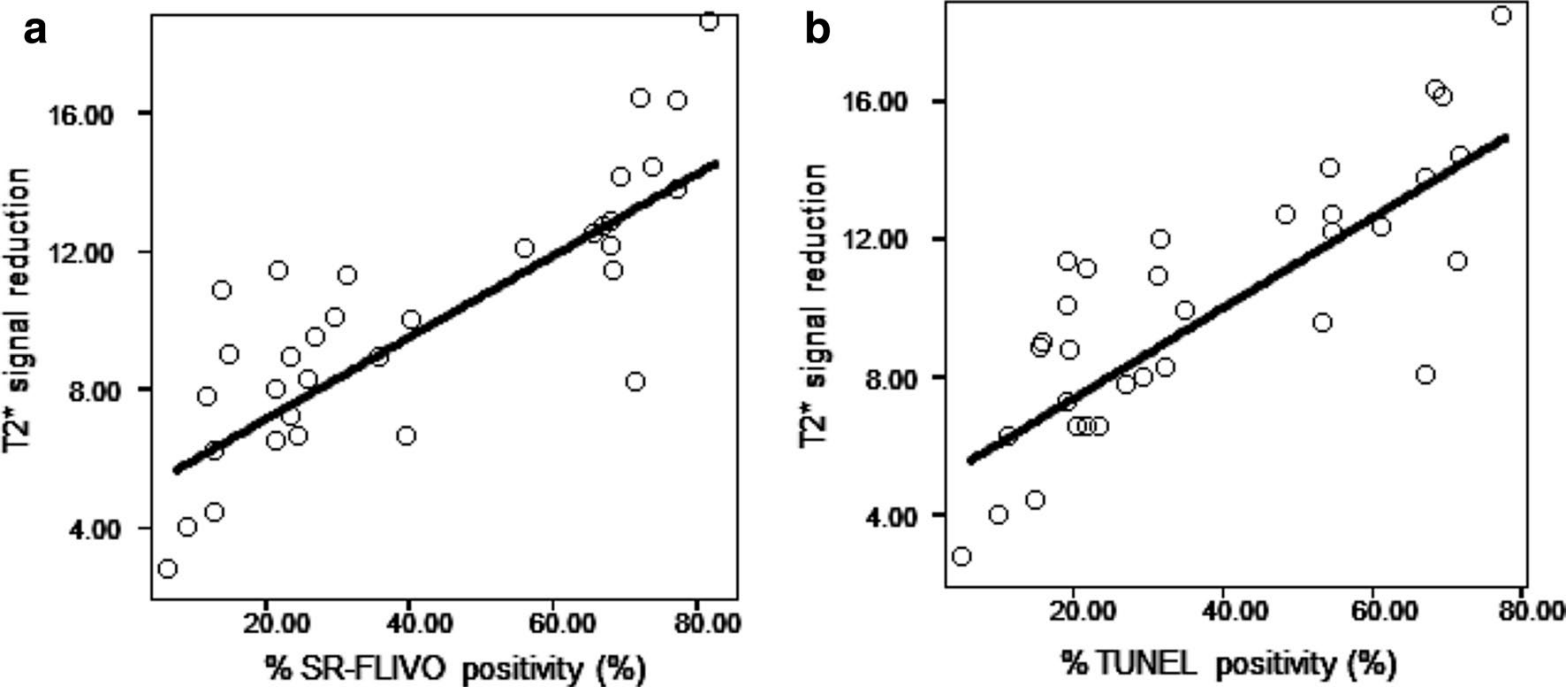
Statistical data of correlation of T2* signal reduction with ratio of SR-FLIVO positivity and TUNEL positivity after SR-FLIVO-MNP administration. Linear relationships were observed between T2* signal reduction and SR-FLIVO (T2* reduction = 4.85 + 0.12 × % FLIVO positivity, *n* = 33, r = 0.818, *P* < 0.0001) (**a**). Linear relationships were observed between T2* signal reduction and TUNEL positivity (T2* reduction = 4.77 + 0.12 × % TUNEL positivity, *n* = 33, r = 0.801, *P* < 0.0001) (**b**)

**Fig. 7:**
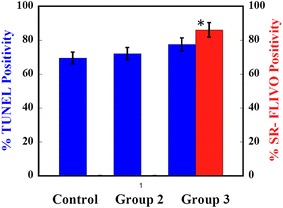
Histological statistical analysis of average % TUNEL positivity and % SR-FLIVO positivity estimated from ischemic rat brain tissues, control, FMNP injected rats (*group 2*) and SR-FLIVO-FMNP injected rats (*group 3*). Significant average positivity of SR-FLIVO-FMNP injected group was observed compared with that in the bare FMNP injected and control groups (*P < 0.003)

We histologically examined the occurrence of apoptosis and iron accumulation in the ROI for the T2* reduction area after administration of SR-FLIVO-FMNP. No iron positivity was observed in the non-ischemic lesion. Electron microscopic findings showed intracellular SR-FLIVO-FMNP deposition in apoptotic cells (Fig. 5). These findings of intracellular SR-FLIVO-FMNP deposition were morphologically accordant with a previous report [50]. Intracellular SR-FLIVO expressions were prominently observed in TUNEL-positive cells in the ROI (Fig. 5). The frequency of SR-FLIVO or TUNEL immunopositivity was calculated in the ROI in the T2* signal reduction area. Correlation of the ratios of immunopositivity and quantitative data of T2* signal reduction was analyzed (Fig. 6). The statistical evaluation revealed a significant linear correlation of T2* signal reduction to the ratio of SR-FLIVO immunopositive cells per square in the ROI (*n* = 33, r = 0.818, *P* < 0.0001) (Fig. 6). T2* signal reduction was also significantly correlated to the ratio of TUNEL immunopositivity per square in the ROI (*n* = 33, r = 0.801, *P* < 0.0001) (Fig. 6). The average quantified distribution of positive % SR-FLIVO and % positive TUNEL was plotted for the three studied groups (control, FMNP and SR-FLIVO-FMNP injected ischemic rats). Significant positive distribution was found and co-localized with apoptotic positive TUNEL cells located within the ischemic lesion at rat brain tissues for the SR-FLIVO-FMNP injected group (Fig. 7). Table 1 summarizes the statistical data correlated to the histological evaluation. Significant co-localization percentage of 69.53 ± 9.95 suggests that; SR-FLIVO-FMNP probe could be used in addition to TUNEL staining protocol for the in/ex vivo apoptotic cells detection.

**Table 1:** Statistical analysis of % TUNEL positivity, SR-FLIVO positivity and their co-localization estimated for control, FMNP injected and SR-FLIVO-FMNP injected ischemic rat groups

	Group 1 (control)	Group 2 (FMNP injected rats)	Group 3 (SR-FLIVO-FMNP injected rats)
% TUNEL positivity	69.46 ± 8.78	72.08 ± 9.06	78.83 ± 10.22
% SR-FLIVO positivity	–	–	86.50 ± 9.11
% Co-localized	–	–	69.53 ± 9.95

SR-FLIVO-FMNP probe was newly developed by conjugation of −NH_2_ functionalized Fe_2_O_3_ MNP at silica to SR-FLIVO [31]. Real system applicability of SR-FLIVO-FMNP probe in the development of neuroradiological evaluation method could be achieved.

We demonstrated the following findings for the first time: (1) SR-FLIVO-FMNP was intravenously administered into ischemic rats and FLIVO expression was found in apoptotic cells in rat brains after cerebral ischemia. (2) Injection of SR-FLIVO-FMNP lead to a remarkable T2* reduction in the ischemic brain, however, bare FMNP administration did not show a T2* reduction on MRI subtraction. (3) SR-FLIVO-FMNP probe accumulation was observed in apoptotic cells in the region of the T2* reduction and almost no probe accumulation were observed in non-apoptotic cells. (4) The findings of T2* reduction on MRI subtraction in ischemic rat brains were significantly correlated to immunopositivities of TUNEL and FLIVO expression. Histological analysis showed that almost all FLIVO-positive cells were positive for TUNEL staining. The small discrepancy between FLIVO and TUNEL might be due to the difference in peak time points of activated caspases and DNA fragmentation. Iron accumulation after intravenous injection was non-specifically observed in the regions of vascular–rich tissue such as choroid plexus and inflammatory lesion [37]. The T2* reduction area in MRI included the contralateral paraventricular region to a small extent. However, our results showed limited non-specific accumulation. This could reflect the surface modification of iron oxide MNPs coated with functionalized silica shell, which might produce better biostability and contribute to less non-specific adhesion. The clear mechanisms of metabolism and circulatory dynamics after intravenous injection of SR-FLIVO-FMNP were of interest as additional features. Our new findings shed the light on a new conjugated nanomaterial for MRI detection of apoptosis. These findings suggest the possibility of targeting delivery to live apoptotic cells and could contribute to the establishment of future methods for in vivo molecular imaging and noninvasive targeted delivery.

## Conclusions

SR-FLIVO-FMNP was successfully delivered to apoptotic cells in ischemic brains after intravenous administration and was shown to be an effective tool for MRI detection of in vivo apoptosis. Targeting delivery with FMNPs conjugated with an enzyme or antibody might be effective for molecular MRI and for the development of a new cellular therapeutic strategy with high specificity. Multifunctional SR-FLIVO-FMNP has possibilities as a noninvasive imaging agent, for specific drug delivery and for intracellular tracking of targeting cells or administered agents for live apoptotic lesions. Safe and effective multifunctional MNPs might contribute to molecular diagnosis at clinical examination and be highly targeted therapeutic tools in the future.

## Additional file

10.1186/s12951-016-0173-1 Phantom MRI of conjugated SR-FLIVO-FMNP showing T2 weighted signal reduction based on Fe concentration that was determined using Shimadzu atomic absorption spectrphotometer AA-6200.
