# Supplementary material for: Noninvasive targeting delivery and in vivo magnetic resonance tracking method for live apoptotic cells in cerebral ischemia with functional Fe2O3 magnetic nanoparticles
Source: J Nanobiotechnology. 2016 Mar 11;14:19. doi: 10.1186/s12951-016-0173-1 (PMC4788935; doi:10.1186/s12951-016-0173-1)
Supplement: Supplementary file 1 — 10.1186/s12951-016-0173-1 Phantom MRI of conjugated SR-FLIVO-FMNP showing T2 weighted signal reduction based on Fe concentration that was determined using Shimadzu atomic absorption spectrphotometer AA-6200. [file 12951_2016_173_MOESM1_ESM.docx]

**Supporting Information**

## **Noninvasive targeting delivery and in vivo magnetic resonance tracking method for live apoptotic cells in cerebral ischemia with functional Fe_2_O_3_ magnetic nanoparticle**

## **Atsushi Saito^1, 2*^, Moataz M. Mekawy^2*^, Akira Sumiyoshi^3^, Jorge J. Riera^3^, Hiroaki Shimizu^2^, Ryuta Kawashima^3^, and Teiji Tominaga^2^**

## ^1^Department of Neurosurgery, Aomori Prefectural Central Hospital, Aomori, Japan; ^2^Department of Neurosurgery, Tohoku University, Graduate School of Medicine, ^3^Department of Functional Brain Imaging, Institute of Development, Aging and Cancer, Tohoku University, Sendai, Japan.

**T2 (ms)**

**Figure S1.** Phantom MRI of conjugated SR-FLIVO-FMNP showing T2 weighted signal reduction based on Fe concentration that was determined using Shimadzu atomic absorption spectrophotometer AA-6200.

**Figure S2.** Surface zeta potential of SR-FLIVO-FMNP probe which shows a slightly negative surface charge.
